# High-frequency aberrantly methylated targets in pancreatic adenocarcinoma identified via global DNA methylation analysis using methylCap-seq

**DOI:** 10.1186/1868-7083-6-18

**Published:** 2014-09-22

**Authors:** Yangxing Zhao, Jinfeng Sun, Hongyu Zhang, Shicheng Guo, Jun Gu, Wei Wang, Ning Tang, Xiaoyu Zhou, Jian Yu

**Affiliations:** 1 State Key Laboratory of Oncogenes and Related Genes, Shanghai Cancer Institute, Renji Hospital, Shanghai Jiao Tong University School of Medicine, LN 2200/25, Xietu Road, Shanghai 200032, China; 2 Zhongshan Hospital, Fudan University, Shanghai, China; 3 Shanghai Cancer Institute, Renji Hospital, Shanghai Jiao Tong University School of Medicine, Shanghai, China; 4 Ministry of Education Key Laboratory of Contemporary Anthropology School of Life Sciences, Fudan University, Shanghai, China; 5 Key Laboratory of Contraceptive Drugs and Devices of NPFPC, Shanghai Institute of Planned Parenthood Research, Shanghai, China

**Keywords:** CGI shore, DNA methylation, genome-wide, methyl capture sequencing, orphan CGI, pancreatic adenocarcinoma

## Abstract

**Background:**

Extensive reprogramming and dysregulation of DNA methylation is an important characteristic of pancreatic cancer (PC). Our study aimed to characterize the genomic methylation patterns in various genomic contexts of PC. The methyl capture sequencing (methylCap-seq) method was used to map differently methylated regions (DMRs) in pooled samples from ten PC tissues and ten adjacent non-tumor (PN) tissues. A selection of DMRs was validated in an independent set of PC and PN samples using methylation-specific PCR (MSP), bisulfite sequencing PCR (BSP), and methylation sensitive restriction enzyme-based qPCR (MSRE-qPCR). The mRNA and expressed sequence tag (EST) expression of the corresponding genes was investigated using RT-qPCR.

**Results:**

A total of 1,131 PC-specific and 727 PN-specific hypermethylated DMRs were identified in association with CpG islands (CGIs), including gene-associated CGIs and orphan CGIs; 2,955 PC-specific and 2,386 PN-specific hypermethylated DMRs were associated with gene promoters, including promoters containing or lacking CGIs. Moreover, 1,744 PC-specific and 1,488 PN-specific hypermethylated DMRs were found to be associated with CGIs or CGI shores. These results suggested that aberrant hypermethylation in PC typically occurs in regions surrounding the transcription start site (TSS). The BSP, MSP, MSRE-qPCR, and RT-qPCR data indicated that the aberrant DNA methylation in PC tissue and in PC cell lines was associated with gene (or corresponding EST) expression.

**Conclusions:**

Our study characterized the genome-wide DNA methylation patterns in PC and identified DMRs that were distributed among various genomic contexts that might influence the expression of corresponding genes or transcripts to promote PC. These DMRs might serve as diagnostic biomarkers or therapeutic targets for PC.

## Background

Pancreatic cancer (PC), a highly malignant tumor of the digestive system, is a type of solid tumor that currently has one of the worst prognoses, with a postoperative 5-year survival rate of less than 25%. Nearly 100,000 people die from PC every year in the USA and Europe; PC ranks fourth and fifth for cancer mortality in those countries, respectively [1]. The incidence of PC in China is also displaying an increasing trend [2].

From a biological perspective, genetics is predominantly responsible for the stable transfer of hereditary information between generations, whereas the distinct somatic phenotypes in different tissues and cells are influenced by epigenetics. Because most tumors that develop display particular acquired biological phenotypes, epigenetic changes must surely play important roles during tumor development [3]. DNA methylation, a well-studied epigenetic phenomenon, has already been extensively studied in PC. At the gene particular level, genes such as *p14ARF* and *p16INK4a*[4,5] were found to display aberrant promoter methylation in PC, leading to abnormalities in gene transcription. At the genomic level, by combining the techniques of methylated CGI amplification with Agilent 244 K Human Promoter ChIP-on-chip microarrays, the genome-wide methylation abnormalities in PC have been identified [6,7].

However, methylation array technology platform-based studies are typically focused on CpG islands (CGIs) and presumably provide less coverage of the entire genome than studies using next-generation sequencing technology [8]. Therefore, the details of the genome-wide methylation profile of PC reported by these studies should be supplemented further, especially in regions such as CGI shores (2 kb regions flanking a CGI), non-CGI promoter regions, and non-gene-associated CGIs (orphan CGIs), as it has already been suggested that methylation changes in these particular regions are associated with certain tumor phenotypes or with tissue specificity [9,10].

In light of these next-generation sequencing technologies, the methylated portion of the genome identified using methyl capture sequencing (methylCap-seq) [11] has been profiled in greater detail than using an array-based platform, revealing many novel regions that are differently methylated in a biological sample. Here, we report a comparison of global DNA methylation patterns between pooled PC tissue and pooled adjacent (PN) tissue samples to identify the critical epigenetic effectors responsible for the malignant phenotype of PC. Our study characterized the genome-wide methylation profile of PC and identified the genomic regions displaying a high frequency of aberrant methylation, including regions of gene-associated CGIs, orphan CGIs, CpG shores, and gene promoters lacking CGIs. The aberrant DNA methylation that occurred in these regions was separated into two categories: aberrant DNA methylations that downregulated gene expression, and those that did not affect gene expression. The former category might be related to a tumorigenesis mechanism; thus, these methylations should be studied biofunctionally and might represent targets for tumor treatment, and the latter methylations may be considered potential biomarkers for PC diagnosis.

## Results

### Wide-spread aberrant hypermethylation in PC and PN revealed via genomic methylation profiling

**Figure 1:**
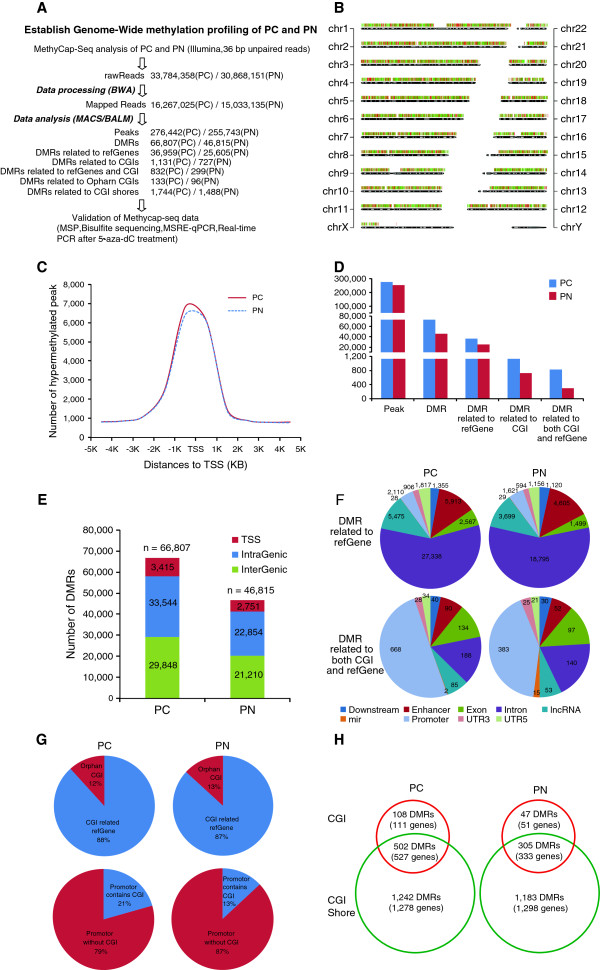
**Data mining of MethylCap-seq libraries. (A)** Experimental strategy to evaluate differential DNA methylation in PC compared with PN. **(B)** Chromosomal view of genome-wide distribution of hypermethylated DNA in PC compared with PN. Red bar, hypermethylation in PC; green bar, hypermethylation in PN. **(C)** Hypermethylated peaks around the TSS site in PC compared with those in PN. Peaks were surveyed in a broad region (from 5 kb downstream to 5 kb upstream of the TSS). **(D)** Mapping peaks and differently methylated regions (DMRs) that were specific for PC and PN. The DMRs are shown according to their inclusion in different gene structure context, such as refGene or CpG island (CGI) definitions. Note that the *y*-axis is interrupted to show whole dataset. **(E)** Genomic distribution of DMRs with PC and PN in transcription start sites (TSSs), intragenic regions, and intergenic regions. The total number of DMRs is presented at the top of each graph. **(F)** DMR distribution over the various gene structures based on sole refGene involvement versus both CGI and refGene involvement in PC-specific and PN-specific DMRs. The genomic context is defined as that found in the UCSC database. **(G)** PC- and PN-selective DMR distribution over orphan CGIs versus refGene-related CGIs, and over CGI-containing promoters vs. no-CGI promoters. **(H)** DMRs (and their related genes) in PC and PN, considering the involvement of various CGI features (CGI, CGI shore, or both). CGI, CpG island; DMR, differently methylated regions. PC, pancreatic cancer; PN, adjacent non-tumor tissue; TSS, transcription start site.

The clinical characteristics of the patients enrolled in this study are listed in Table 1. The whole-genome methylation profiles of the PC and PN samples were successfully identified using the methylCap-seq method. Exogenous fully methylated and unmethylated spike DNA fragments were used as controls to confirm the capture accuracy of hypermethylated DNA fragments (Additional file 1: Figure S1). We acquired 33,784,358 raw reads in the PC group and 30,868,151 raw reads in the PN group. Based on their alignment with the human genome (hg19) sequence, 16,267,025 (48.15%) raw reads in the PC group and 15,033,135 (48.70%) raw reads in the PN group were uniquely positioned. The reads mapping to 28,691 CGIs, which were defined using the University of California, Santa Cruz (UCSC) Genome Browser, were investigated: 3.57% of the reads in the PC group and 4.25% of the reads in the PN group were positioned at CGIs, resulting in a CGI coverage rate of 64.31% in the PC group and 64.36% in the PN group. These data indicated that our experiment provided considerable information regarding genomic CGIs (Figure 1A).Accumulation of the mapped reads formed peaks. In total, 276,442 and 255,743 peaks were found in the PC and PN samples, respectively, displaying distinct distributions of hypermethylated regions between PC and PN throughout the chromosomes (Figure 1B). An analysis of the hypermethylated peaks approximately 5 kb from the transcription start site (TSS) revealed that methylation peaks accumulated near TSSs, and that more of these peaks were detected in the PC group than in the PN group (Figure 1C). After removing the peaks common to both PC and PN samples (approximately 209,000), 66,807 PC-specific and 46,815 PN-specific hypermethylated differently methylated regions (DMRs) were identified (Figure 1D). Of these hypermethylated DMRs, 36,959 PC-specific and 25,605 PN-specific DMRs were located within genes (Figure 1E), and 1,131 hypermethylated DMRs in PC tissue and 727 hypermethylated DMRs in PN tissue were associated with CGIs. The hypermethylated DMRs were separated into three categories: TSS, intragenic or intergenic (Figure 1E). Subsequently, the location of the TSS DMRs and intragenic DMRs were further determined using structural annotations of the human genome, such as downstream, enhancer, exon, intron, miRNA, promoter, and 5′ UTR (Figure 1F).

**Table 1:** Clinical profile of the PC patients recruited in this study

		**Methylome group**		***P***	**Test group**		***P***
		**Pancreatic carcinoma**	**Non-tumor tissue adjacent to pancreatic carcinoma**		**Pancreatic carcinoma**	**Non-tumor tissue adjacent to pancreatic carcinoma**	
Number of patients		10	10		16	15	
Sex							
	Male	6 (60.0%)	6 (60.0%)	1.000	12 (75.0%)	12 (75.0%)	0.638
	Female	4 (40.0%)	4 (40.0%)		4 (25.0%)	4 (25.0%)	
Age							
	>60 years	7 (70.0%)	7 (70.0%)	1.000	12 (75.0%)	12 (80.0%)	1.000
	≤60 years	3 (30.0%	3 (30.0%		4 (25.0%)	3 (20.0%)	
Tumor location							
	Head	7 (70.0%)	7 (70.0%)	1.000	13 (71.2%)	13 (86.7%)	1.000
	Body and tail	3 (30.0%	3 (30.0%		3 (18.8%)	2 (13.3%)	
Differentiation							
	Poor	4 (40.0%)	4 (40.0%)	1.000	3 (37.5%)	1 (20.0%)	1.000
	Moderate	6 (60.0%)	6 (60.0%)		5(63.5%)	4 (80.0%)	
	High	0 (0.0%)	0 (0.0%)		0 (0.0%)	0 (0.0%)	
Tumor stage							
	I, II	7 (70.0%)	7 (70.0%)	1.000	6 (75.0%)	4 (80.0%)	1.000
	III, IV	3 (30.0%)	3 (30.0%)		2 (25.0%)	1 (20.0%)	
Tumor size							
	≤3 cm	6 (60.0%)	6 (60.0%)	1.000	6 (75.0%)	4 (80.0%)	0.638
	>3 cm	4 (40.0%)	4 (40.0%)		2 (25.0%)	1 (20.0%)	
Lymph node metastasis							
	Yes	8 (80.0%)	8 (80.0%)	1.000	7 (87.5%)	4 (80.0%)	1.000
	No	2 (20.0%)	2 (20.0%)		1 (12.5%)	1 (20.0%)	

This analysis yielded 1,859 DMRs that mapped to CGIs in both the PC and PN samples, and these DMRs represented the vast majority of the hypermethylated CGIs in the refGene category for PC (88%) and PN (87%); orphan CGIs, which are not associated with any known refGene, accounted only for 12% (133 orphan CGIs of 1,131 affected CGIs; Additional file 2: Table S1) and 13% (96 orphan CGIs of 727 affected CGIs; Additional file 3: Table S2) of the CGI-related DMRs in PC and PN tissue, respectively (Figure 1G). There was no difference in the frequency of orphan CGIs among the DMRs between the PC and PN samples. When the gene-associated DMRs were considered, 5,341 such DMRs were identified; we found a higher frequency of CGI-containing promoters in PC tissue (21%, 609/2,955; Additional file 4: Table S3) than in PN tissue (13%, 312/2,386; Additional file 5: Table S4) (Figure 1G), suggesting the importance of CGIs in aberrant DNA methylation during PC tumorigenesis.

It is generally accepted that methylation of a CGI within a promoter is responsible for gene expression silencing. However, two recent studies discovered that certain types of tumor- and tissue-specific methylation occur in CGI shores that play important biological roles. In this study, aside from examining the methylation status of CGIs, we investigated the methylation status of CGI shores, particularly those associated with gene promoters. Regarding the hypermethylated genes and their sites of hypermethylation (in the CGI only, the CGI shore only, or both) in PC tissue, 527 genes (Additional file 6: Table S5) were hypermethylated in a CGI and a CGI shore (502 DMRs); 111 genes (Additional file 7: Table S6) were hypermethylated in a CGI alone (108 DMRs), and 1,278 genes (Additional file 8: Table S7) were hypermethylated in a CGI shore alone (1.242 DMRs). In contrast, based on analysis of the hypermethylated genes and sites of hypermethylation in PN tissue, 333 genes (Additional file 9: Table S8) were hypermethylated in both a CGI and a CGI shore (305 DMRs), 51 genes (Additional file 10: Table S9) were hypermethylated in a CGI alone (47 DMRs), and 1,298 genes (Additional file 11: Table S10) were hypermethylated in a CGI shore only (1,183 DMRs) (Figure 1H). In general, the number of genes aberrantly methylated in a CGI was much fewer, accounting for 20% to 25% of the total genes affected. More frequently, the genes were methylated in a CGI shore, which is probably due to the extended genomic regions defined by the CGI shore. We detected more abnormally hypermethylated genes in a CGI in PC tissue than in PN tissue (*P* = 0.0002, Chi-square test), suggesting that CGIs are more likely to contain an aberrant DNA methylation target during PC development.

### Gene ontology (GO) and Kyoto Encyclopedia of Genes and Genomes (KEGG) pathway analysis of the aberrantly methylated genes in PC

**Figure 2:**
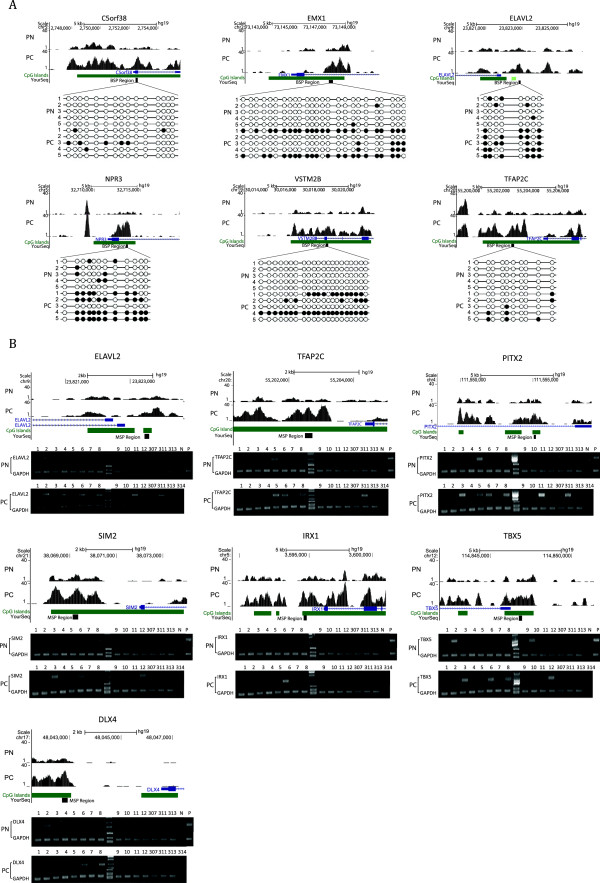
**Representative results of bisulfite sequencing PCR (BSP) and methylation-specific PCR (MSP) validation of methylCap-seq data.** For each gene, the UCSC scheme of the gene locus and the examined promoter regions are shown. **(A)** BSP results. **(B)** MSP results. 15 pairs of PC and PN samples (1,2,3,4,5,6,7,8,9,10,11,12, 307,311,313) and an extra 1 PC (314) were evaluated. All the samples were assayed by MSP. GAPDH: GAPDH-BSP were amplified as quality and quantity control for the confirmation of bisulfite-converted DNA templates. N, negative control; P, positive control; PC, pancreatic cancer; PN, adjacent non-tumor tissue.

It is well accepted that methylation abnormalities within promoters can influence the expression of the corresponding genes. Therefore, we conducted GO analysis of the genes that displayed promoter hypermethylation in PC and PN tissue. A significance of *P* < 0.05 indicated gene enrichment in several GO categories (Table 2). We determined that 668 hypermethylated genes in PC tissue (Additional file 12: Table S11) were enriched in ‘sequence-specific DNA binding’ (GO:0043565), ‘neuron differentiation’ (GO:0030182), ‘regulation of transcription, DNA-dependent’ (GO:0006355), or ‘cell morphogenesis involved in differentiation’ (GO:0000904) and that 383 hypermethylated genes in PN tissue (Additional file 13: Table S12) were enriched in ‘plasma membrane part’ (GO:0044459), ‘channel regulator activity’ (GO:0016247), ‘positive regulation of bone morphogenetic protein (BMP) signaling pathway’ (GO:0030513), ‘protein homo oligomerization’ (GO:0051260), or ‘neuron differentiation’ (GO:0030182). Furthermore, we identified 111 genes containing hypermethylated promoters in PC that were enriched in ‘regulation of transcription term’; among these genes, the methylation status of *DLX4*, *ELAVL2*, *IRX1*, *PITX2*, *SIM2*, *TBX5*, and *TFAP2C* was subsequently validated in the tissue samples by methylation-specific PCR (MSP) (Figure 2B).

**Table 2:** Gene ontology enrichment analysis of aberrant methylation in gene promoters in PC and PN

	**Category**	**Term**	**Genes in list**	**Total genes**	-log10 ( *P *)*
Hypermethylated promoter related gene (615)	GOTERM_MF_FAT	GO:0043565 sequence-specific DNA binding	61	607	8.47 × 10^-16^
	GOTERM_BP_FAT	GO:0030182 neuron differentiation	47	438	5.06 × 10^-13^
	GOTERM_BP_FAT	GO:0006355 regulation of transcription, DNA-dependent	111	1773	5.08 × 10^-13^
	GOTERM_BP_FAT	GO:0000904 cell morphogenesis involved in differentiation	29	244	3.07 × 10^-9^
	GOTERM_BP_FAT	GO:0006928 cell motion	41	475	1.30 × 10^-8^
	GOTERM_BP_FAT	GO:0007267 cell-cell signaling	47	600	1.88 × 10^-8^
	GOTERM_CC_FAT	GO:0043005 neuron projection	31	342	4.13 × 10^-8^
	GOTERM_CC_FAT	GO:0045202 synapse	28	355	3.28 × 10^-6^
	GOTERM_CC_FAT	GO:0044459 plasma membrane part	96	2203	9.52 × 10^-6^
	GOTERM_CC_FAT	GO:0005887 integral to plasma membrane	60	1188	1.40 × 10^-5^
	GOTERM_CC_FAT	GO:0044456 synapse part	21	246	2.43 × 10^-5^
	GOTERM_MF_FAT	GO:0022836 gated channel activity	24	310	1.03 × 10^-4^
	GOTERM_MF_FAT	GO:0005021 vascular endothelial growth factor receptor activity	4	8	1.48 × 10^-3^
	GOTERM_MF_FAT	GO:0003702 RNA polymerase II transcription factor activity	18	244	1.61 × 10^-3^
	GOTERM_MF_FAT	GO:0030955 potassium ion binding	12	128	2.12 × 10^-3^
	KEGG_PATHWAY	hsa04080 neuroactive ligand-receptor interaction	17	256	3.33 × 10^-3^
Hypomethylated promoter related gene (383)	GOTERM_CC_FAT	GO:0044459 plasma membrane part	63	2203	2.05 × 10^-5^
	GOTERM_MF_FAT	GO:0016247 channel regulator activity	6	59	3.00 × 10^-3^
	GOTERM_BP_FAT	GO:0030513 positive regulation of BMP signaling pathway	3	6	4.06 × 10^-3^
	GOTERM_BP_FAT	GO:0051260 protein homooligomerization	7	95	5.36 × 10^-3^
	GOTERM_BP_FAT	GO:0030182 neuron differentiation	16	438	7.41 × 10^-3^
	GOTERM_BP_FAT	GO:0048732 gland development	8	135	7.90 × 10^-3^
	GOTERM_BP_FAT	GO:0031328 positive regulation of cellular biosynthetic process	21	685	1.19 × 10^-2^
	GOTERM_CC_FAT	GO:0005902 microvillus	4	36	2.28 × 10^-2^
	GOTERM_CC_FAT	GO:0032420 stereocilium	3	15	2.60 × 10^-2^
	GOTERM_CC_FAT	GO:0032421 stereocilium bundle	3	17	3.30 × 10^-2^
	GOTERM_MF_FAT	GO:0016524 latrotoxin receptor activity	2	2	3.32 × 10^-2^
	GOTERM_CC_FAT	GO:0005923 tight junction	5	73	3.54 × 10^-2^
	GOTERM_MF_FAT	GO:0003700 transcription factor activity	25	975	3.60 × 10^-2^
	GOTERM_MF_FAT	GO:0005261 cation channel activity	10	275	4.12 × 10^-2^

*Logarithmic transformation of *P* to show significance level of the differential methylation region (in differently methylated region (DMR) estimation) or methylation blocks (model-based analysis of ChIP-Seq). The higher this value, the higher the probability inferred for DMR or methylation blocks.

A KEGG pathway analysis of the aforementioned genes revealed that hypermethylated genes in both PC and PN tissue were enriched in ‘neuroactive ligand-receptor interaction’ (hsa04080) (Table 2). Furthermore, several miRNAs were aberrantly methylated in PC tissue. The PC-related hypermethylated miRNAs included mir-9-3, mir-9-1, mir-124-3, mir-10b, mir-124-2, mir-718, and mir-203; the PN-related hypermethylated miRNAs included mir-210, mir-1469, mir-130b, mir-149, mir-1224, and mir-564 (Table 3).

**Table 3:** Aberrantly hypermethylated miRNAs in PC and PN in present and previous studies

	**Names**	**Differently methylated region location in genome**	-10log ( *P *)*	**Transcription direction**	**References**
**Hypermethylated**	hsa-mir-9-3	chr15:89908727-89909052	78.11	Sense	[24]
	hsa-mir-9-1	chr1:156389946-156390692	81.02	Antisense	[25]
	hsa-mir-124-3	chr20:61806943-61807560	30.36	Sense	[21]
	hsa-mir-10b	chr2:177013457-177013940	366.4	Sense	[28,29]
	hsa-mir-124-2	chr8:65289087-65292713	387.77	Sense	[20,26]
	hsa-mir-718	chrX:153284470-153285204	65.44	Antisense	
	hsa-mir-203	chr14:104584097-104584701	92.85	Sense	[27]
**Hypomethylated**	hsa-mir-210	chr11:569133-569765	95.42	Antisense	[23]
	hsa-mir-1469	chr15:96877281-96877871	81.68	Sense	
	hsa-mir-130b	chr22:22006547-22007358	143.05	Sense	[22]
	hsa-mir-149	chr2:241395079-241395714	58.15	Sense	
	hsa-mir-1224	chr3:183958940-183960006	126.15	Sense	
	hsa-mir-564	chr3:44902453-44903064	46.02	Sense	

*Logarithmic transformation of P to show significance level of the differential methylation region (in differently methylated region (DMR) estimation) or methylation blocks (model-based analysis of ChIP-Seq). The higher this value, the higher the probability inferred for DMR or methylation block.

We analyzed the hypermethylated genes that were enriched in PC and PN tissue, focusing on their functional involvement in tumorigenesis, and found that the aberrantly methylated genes could be categorized as either tumor promoters or tumor suppressors. The corresponding genes included 20 tumor promoters and 10 tumor suppressors among the PC-related hypermethylated genes and 10 tumor promoters and 5 tumor suppressors among the PN-related hypermethylated genes (Additional file 14: Table S13). These results suggest that aberrant DNA methylation plays an important role in tumor development via the important biological pathways related to the regulation of tumorigenesis.

### Verification of PC-specific DMRs identified in methylCap-seq

The accuracy and precision of the DMR profiles were validated in two sample sets, which consisted of the pooled samples used to generate the methylCap-seq library and another independent sample set. In one validation vignette, the DNA methylation status of the ten most significant DMRs that were located in a promoter region (*P* <10^-15^) were evaluated using bisulfite sequencing PCR (BSP) in the same set of PC and PN samples used to generate the methylCap-seq libraries. Six candidate DMR genes, *C5orf38*, *EMX1*, *NPR3*, *VSTM2B*, *ELAVL2*, and *TFAP2C*, were validated to be significantly hypermethylated in PC tissue compared with PN tissue using the BSP technique (representative results are shown in Figure 2A).

### Preliminary detection of the DMRs identified by genome methylation profiling in limited clinical PC samples

In another validation vignette, the methylation status of 20 gene-associated DMRs scattered throughout various genetic elements, such as promoters, miRNAs, introns, exons, and CGI shores, were analyzed using MSP in paired samples of PC and PN tissue. The results revealed that seven gene-associated DMRs displayed clear differences in methylation between the PC and PN samples (representative results are shown in Figure 2B). The DMRs in the promoter regions of *TRADD*, *AGAP2*, and *FAM115A* displayed a loss of methylation in PC tissue (The MSP results are presented in Additional file 15: Table S14).

### MSRE-qPCR and RT-qPCR validation of the methylation of orphan CGIs and the expression of corresponding ESTs in PC cell lines treated with 5-aza-2′-deoxycytidine (5-aza-dc)

**Figure 3:**
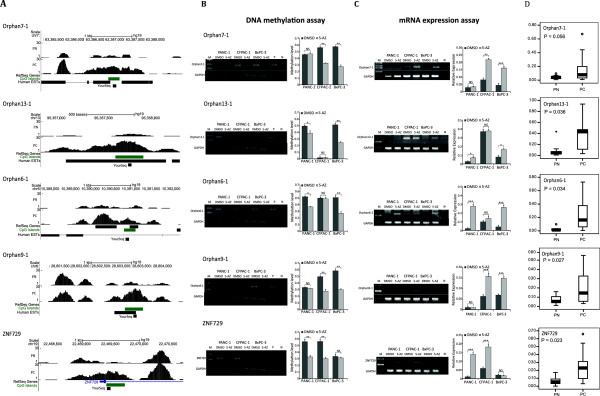
**Methylation of CGIs (orphan CGIs or regular CGIs) might influence the expression of putative ESTs or mRNAs. (A)** UCSC scheme of CGIs and the nearby putative ESTs or mRNAs analyzed in this study. **(B)** DNA methylation changes in PC cell lines after treatment with 5-aza-dc. The GAPDH-BSP product serves as a quality and quantity control for the bisulfite-converted DNA templates. **(C)** EST expression after 5-aza-dc treatment determined by RT-qPCR. GAPDH mRNA expression was the loading control. **(D)** Quantitative analysis of DNA methylation by methylation sensitive restriction enzyme-based qPCR (MSRE-qPCR) in eight PC and five PN samples. The box is defined by 25% and 75% quantiles. The methylation levels in the PC and PN samples were compared by one-way analysis of variance (ANOVA), and the *P* values are indicated. 5-AZ, 5-aza-2′-deoxycytidine; DMSO, dimethyl sulfoxide; EST, expressed sequence tag; N, negative control; P, positive control; PC, pancreatic cancer; PN, PN, adjacent non-tumor tissue.

Three PC cell lines were treated with 5-aza-2′-deoxycytidine (5-aza-dc). The methylation status of ten hypermethylated DMRs (for gene locus information, see Figure 3A) in promoter CGIs and orphan CGIs was quantitatively analyzed via methylation sensitive restriction enzyme-based qPCR (MSRE-qPCR) (Figure 3B) in these three PC cell lines before and after treatment with 5-aza-dc. The expression levels of gene-associated CGI-containing genes and orphan CGI-containing expressed sequence tags (ESTs) were analyzed using RT-qPCR to ascertain the correlation between the aberrant DMRs and the corresponding mRNA expression levels (Figure 3C). The results indicated that the methylation levels of four orphan CGIs and one promoter CGI were decreased and that the mRNA expression of the corresponding genes or ESTs increased upon 5-aza-dc treatment, suggesting that the expression of these genes or ESTs might be regulated by DNA methylation. Quantitative analysis of the methylation status of these particular DMRs in an independent set of samples (testing group: eight PC samples, five PN samples, and three PC cell lines) confirmed the differences in methylation at these four DMRs in the clinical samples (Figure 3D).

## Discussion

Pooling strategies have been utilized in many previous genomic studies to investigate the phenotypic similarities between specific models, owing to the advantage of conserving samples [12,13]. In this study, genome-wide methylation profiles of PC and PN tissues were established using corresponding pooled samples.

We identified 5,280 and 3,488 hypermethylated DMRs in PC and PN tissue, respectively, that were closely associated with genes and CGIs. Gene ontology analysis of the genes associated with these DMRs revealed that the aberrantly hypermethylated genes primarily belonged to categories related to nucleic acid binding, DNA binding, and activation of transcription factors, suggesting that the methylation of these affected genes coupled with the downregulation of RNA expression resulted in the decreased expression of other genes. Studies of non-small-cell lung carcinoma by Helman *et al.*[14] and Zhao *et al*. [15] demonstrated that methylation-enriched genes displayed aberrant methylation and RNA expression in multiple tumor types; these genes were referred to as tumor suppressor genes. The methylation-enriched genes in PC associated with ‘cell morphogenesis involved in differentiation’ (GO: 0000904) may participate in the morphological changes and late-stage differentiation in PC tissue. In contrast, the hypermethylated genes in PN tissue were predominantly enriched in ‘plasma membrane part’ (GO:0044459) and ‘channel regulator activity’ (GO:0016247), and it has been confirmed that these genes are enriched in other tumor types in genomic methylation studies, suggesting that abnormal formation of the plasma membrane might be a common characteristic of tumor differentiation and maintenance [16,17].

We also conducted KEGG pathway analysis of the genes containing DMRs in their promoter; surprisingly, the hypermethylated genes in both the PC and PN samples were enriched in the same pathway, ‘neuroactive ligand-receptor interaction’, which primarily participates in the endocrine and exocrine functions of cells. Functional abnormalities in these genes have been demonstrated in studies of meningioma and PC [18,19]. Our study revealed the identical direction of methylation changes in this pathway in PC and PN tissue, but the detailed mechanism by which this pathway participates in PC development requires further investigation (Table 2).

As epigenetic factors, miRNAs play an important role in the regulation of cellular biophysical functions, and have been shown to be associated with the apoptosis, invasion, metastasis, recurrence, and drug resistance of tumor cells. The regulation of miRNAs by DNA methylation has been extensively studied. In this study, we identified particular miRNAs that were aberrantly methylated in PC tissue. Among these miRNAs, hsa-mir-124-3 has been shown to be hypermethylated in PC and is involved in the progression, metastasis, and recurrence of PC [20,21]. Alternatively, mir-130b and mir-210 are two hypermethylated miRNAs in PN tissue that were discovered in this study, both of which were found to be more strongly expressed in PC and have been associated with the proliferation and invasion of PC cells [22,23]. Certain miRNAs that we identified as aberrantly methylated in PC tissue have already been studied in other tumor types and are regulated by DNA methylation. For example, the hypermethylated miRNAs identified in PC tissue, such as miR-9-3, mir-9-1, miR-124, and miR-203, are also hypermethylated in non-small-cell lung carcinoma, breast cancer, cervical cancer, and hematological cancer, respectively; hypermethylation of these sites decreases miRNA expression, which promotes tumor development and tumor cell proliferation [24-27]. In addition, mir-10b, which was found to be hypermethylated in PC tissue in this study, was thought to be an inhibitor of tumor metastasis in animal models [28] and was found to be more strongly expressed in PC [29]. Therefore, further investigation of the remaining miRNAs that were aberrantly methylated in PC will shed light on the mechanisms underlying pancreatic carcinogenesis, as the related research is currently very limited.

The top 40 genes, based on their *P* value, that contained methylation changes in their promoter region were selected and examined using MSP in the test group samples. Of these genes, 18 displayed significant differences in methylation between PC and PN tissues (or PC cell lines). Interestingly, among these genes, seven (*DLX4*, *ELAVL2*, *IRX1*, *PITX2*, *SIM2*, *TBX5*, and *TFAP2C*) were enriched for the annotation of regulation of transcription (GO: 0006355), which corresponds to the results obtained in our previous GO analysis. All of the genes discussed above have been investigated in PC and other tumor types, and their involvement in carcinogenesis has been confirmed.

At both the genetic and genomic levels, many hypermethylated genes previously reported in PC studies were identified as hypermethylated DMRs in the present study, including *LHX1*, *FOXE1*, *PAX6*, *BNIP3*[30], *ALPP*, *CEBPA*[31], *CACNA1G*[32], *CCND2*[33], *BAI1*, *NRN1*, *PENK*, *FAM84A*, and *ZNF415*[6]. In addition, our study identified other genes that are frequently hypermethylated in different types of cancer, such as *RASSF1a*, *CDKN2A*, *hHML1*, and *CDH1*[34,35]. Thus, we have established a relatively extensive database of abnormally methylated sites in PC. We also compared our data with those reported by Omura *et al*. [6], who analyzed nine pairs of PC and PN samples using human CGI microarray 244 k chips, obtaining (after data filtering using the appropriate thresholds) 1,658 differently methylated known loci. This comparison revealed the following. (1) Regarding the ability to capture aberrantly methylated gene targets, methylCap-seq identified more hypermethylated genes than the array method in PC tissue (1983 versus 1206) and PN tissue (1692 versus 379), indicating that methylated DNA fragment enrichment followed by deep sequencing identifies additional aberrant gene loci, although it is more labor-intensive and time-consuming. (2) A total of 737 genes (Additional file 16: Table S15) was identified by both the methylCap-seq and array methods, accounting for 46.7% of the total genes recovered. This high recovery rate between the two methods reflects the reliability of these methods for this purpose. However, the unique genes that were identified suggest that these two methods each have their own particular advantages. (3) The high percentage of commonality among the PC-specific hypermethylated DMR genes (46.2%) and low percentage of commonality among the PN-specific hypermethylated DMR genes (30 genes, 2.9% of all the PN-related hypermethylated DMR genes) (Additional file 17: Table S16) between the study of Omura *et al*. [6] and this study suggests that during the entire process of PC development, hypermethylation is a relatively defined and destined process, whereas hypermethylation at the initiation of tumorigenesis is relatively random or perhaps stochastic.

We also compared our aberrant methylated gene targets determined in this study with the results of previous studies. Among all 3,911 differently methylated genes (DMGs) identified in this study, 728 DMGs were reported by Nourse *et al.*[36], 339 DMGs were reported by Vincent *et al.*[7], and 55 DMGs were reported by Tan *et al*. [37] (Additional file 18: Table S17). This discrepancy in the number of DMGs obtained between the four groups might result from the different technological platforms adopted by each study group, as well as the different ethnic backgrounds of the enrolled patients. Furthermore, this discrepancy emphasizes that the array-based and sequencing-based DNA methylation assay methods must be applied alternately to complement one another, to elucidate DNA methylation at the genomic level.

Deaton and Bird separated CGIs into three categories, TSS, intragenic, and intergenic, with the latter two categories defined as orphan CGIs [38]. Despite poor understanding of the functions of orphan CGIs until recently, studies have shown that orphan CGIs are involved in the regulation of gene transcription, genomic imprinting, and non-coding RNA transcription and that orphan CGIs might display tissue-specific methylation profiles [39]. In this study, we investigated the methylation status of particular orphan CGIs in PC. Hypermethylated orphan CGIs have been found in PC. The methylation status of orphan CGIs was closely associated with the transcription levels of nearby non-annotated ESTs. Further studies should be conducted to clarify whether the methylation-regulated ESTs containing orphan CGIs are potential genes or gene elements. It is well known that merely 6.8% of CpGs are located in CGIs. The methylation status and biological functions of the other 93.2% of CpGs have yet to be adequately studied. The study by Yu *et al.*[40] indicated that the methylation of CpGs in CGI shores is involved in regulating gene transcription or establishing tissue-specific methylation patterns, and changes in the methylation status of CpGs in CGI shores might occur at an earlier stage in carcinogenesis than the changes that occur in gene-associated CGIs. Our study suggests that the methylation changes in all of these CpG regions in PC are indispensable components of the genomic methylation profile of PC and may influence the transcription of PC-related genes and non-coding RNAs, potentially affecting tissue-specific cell differentiation and ultimately leading to carcinogenesis.

It is generally accepted that abnormal DNA hypermethylation can either downregulate gene expression (gene silencing) or exert no influence on gene expression. Gene silencing-related DNA methylation might be involved in PC development, and, therefore, these methylation sites may be examined in tumor development studies and considered as treatment targets. Alternatively, the DNA methylations that are not associated with gene expression might serve as biomarkers of the specific state of PC, and, therefore, these methylation sites could be used for clinical diagnosis. We anticipate that our comprehensive analysis of PC gene methylation will facilitate the further investigation of PC biomarkers for diagnostic, prognostic, and therapeutic applications, by: (1) improving the understanding of the epigenetic importance of DNA methylation in PC tumorigenesis, which might be located outside or within CGIs, including both orphan and gene-associated CGIs; and (2) providing additional candidate targets for PC diagnosis, prognosis and treatment (not limited to the previously reported CGIs and promoters). Moreover, the enormous number of targets (many thousands) obtained suggests a vast heterogeneity among PC patients, thus requiring a large patient cohort for chip-based analyses to validate, optimize, and establish potential targets for ultimate clinical application.

## Conclusions

In the present study, the genome-wide methylation profiles of PC and PN tissues were established using methylCap-seq, revealing globally reprogrammed and deregulated DNA methylation in PC. Compared with PN tissue, there were many PC-specific aberrations in the hypermethylation of CpGs in TSS CGIs, orphan CGIs, CGI shores, and promoter regions lacking CGIs. These findings will be helpful in elucidating the mechanisms underlying pancreatic carcinogenesis related to the DNA methylation-regulated expression of genes and non-coding RNAs. Furthermore, the aberrantly methylated genes in PC identified in this study might serve as potential biomarkers for the early diagnosis and treatment of this deadly disease.

## Methods

### Clinical samples

Pancreatic cancer tissue samples were collected from 18 patients who had undergone surgical treatment without receiving preoperative chemotherapy or radiotherapy from May 2009 to March 2011 in Renji Hospital, School of Medicine, Shanghai Jiao Tong University. A diagnosis of PC was confirmed by histological examination. Resected tumor tissues and matched normal tissues at least 2 cm away from the tumor tissues were collected during the operation, labeled, and stored at -80°C. The sixth edition of the Tumor, Node, and Metastasis (TNM) Staging System proposed by the International Union against Cancer [41] was utilized to stage the tumor tissue samples (Table 1). The study was approved by the medical ethics committee at Renji Hospital, School of Medicine, Shanghai Jiao Tong University. All the patients signed an informed consent form. DNA was isolated from frozen tissues or cell lines using a conventional proteinase K and organic extraction method, as previously described [42].

### Genome-wide methylation profiling by methylCap-seq

Genomic DNA was extracted from ten PC tissues and ten matched normal tissues. Equal amounts of DNA were mixed to form the PC and PN groups. Pooled DNA (1.2 μg) from each group was used to generate the library for methylCap-seq as previously described [42].

### Mapping the sequence reads and DMR identification and annotation

We used Burrows-Wheeler alignment tools [43] with the default settings to map the 36 bp unpaired reads to the hg19 human genome reference assembly [44]. After removing PCR duplicates using Picard, the aligned data were converted, sorted, and indexed using Samtools [45] and Picard [46].

Methylation peaks (hypermethylated regions) were identified using model-based analysis of ChIP-Seq in the PC and PN samples, as previously described [42]. The DMRs between PC and PN were identified using two methods, model-based analysis of ChIP-Seq [47] and a bi-asymmetric-Laplace model (BALM) [48], to increase the detective power of methylCap-seq. To decrease the false positive detection of DMR using BALM, the dual-threshold strategy was applied. A high-confidence threshold (0.975) was utilized in the PC hypermethylated region screening, and a low-confidence threshold (0.950) was utilized in the PN hypermethylated region screening. Cancer-specific methylation peaks were defined as hypermethylated regions. Similarly, normal tissue-specific methylation peaks were defined as hypomethylated regions with the reverse settings. Whole-genome methylation (methylation of each CpG) was inferred using BALM, which was processed for a Pearson correlation analysis among all the samples in the R environment. The refSeq genes (UCSC genes) and corresponding CGIs were downloaded from the table browser of the UCSC database [42]. The browser extensible data (BED) file operations were performed using BEDTools [49] and other Perl scripts. All the scripts are available upon email request. The generated genomic methylation profile was uploaded to a public database (Gene Expression Omnibus: GSE54854). Gene ontology analysis was performed using DAVID Bioinformatics Resources 6.7 [50].

### Methylation analysis

In this study, BSP was utilized to determine the methylation status at single CpG resolution of DMRs identified by genomic methylation profiling; MSP was performed for qualitative methylation screening in a small set of PC samples. Using MSRE-qPCR, the DNA methylation status in orphan CGIs was quantitatively analyzed in PC cell lines before and after 5-aza-2′-deoxycytidine treatment and in small samples of clinical PC tissues, as described previously [51]. Approximately 1.0 μg of genomic DNA extracted from PC or PN samples or PC cell lines was bisulfate-treated using EpiTect Kit (Qiagen, Hilden, Germany). Primers for MSP and BSP were designed using MethPrimer, an online primer design tool [52]. The MSRE-qPCR primers were designed using Primer3 [53]. The sequences of the primers utilized in this study are listed in Additional files 19 and 20: Tables S18 and S19. Jumpstart Taq (Sigma-Aldrich, St. Louis, MO, USA) was used in BSP and MSP with a 20 μl reaction volume per tube. The BSP and MSP reaction conditions were as follows: 94°C for 3 min; 35 cycles of 94°C for 20 s, annealing for 20 s, and 72°C for 20 s; and 72°C for 5 min. The PCR products were analyzed by electrophoresis in 1.5% agarose gels. The PCR products were TA cloned and verified by sequencing. At least five clones were sequenced for each BSP reaction.

### Cell culture and 5-aza-2′-deoxycytidine treatment

Three pancreatic adenocarcinoma cell lines were used: BxPC-3 ATCC, CRL-1687), PANC-1 (ATCC, CRL-1469), and CFPAC-1 (ATCC, CRL-1918). All the cell lines were cultured in RPMI1640 supplemented with 10% FBS, 100 U/ml penicillin, and 100 U/ml streptomycin. All the cell lines were maintained at 37°C in a humidified atmosphere with 5% CO_2_.

The restoration of gene expression by demethylation was evaluated in the BxPC-3, CFPAC-1, and CFPAC-1 cell lines. For the CpG demethylation analysis, exponentially growing cells were seeded at a density of 1.5 × 10^6^ cells/100 mm dish and allowed to attach overnight. The cells were then treated with freshly prepared 5-aza-dC (5.0 μM; Sigma-Aldrich, St. Louis, MO, USA) for 3 days.

### RNA isolation and real-time PCR

Total RNA was prepared from cultured cells using Trizol reagent according to the manufacturer’s instructions (Invitrogen, USA) and then reverse transcribed using an oligo (dT) primer and SuperscriptII RNase H-Reverse Transcriptase (Invitrogen, USA). Real-time PCR was performed with primer pairs for the EST expression assay, and GAPDH was used as the internal control. Real-time PCR was performed as follows: 94°C for 3 min followed by 40 cycles of 94°C for 10 s, 62°C for 10 s, and 72°C for 15 s. Real-time qPCR was performed to detect GAPDH expression with an SYBR Green PCR Kit (Applied Biosystems, Foster city, CA, USA) on a ROTOR-GENE 6000 Real-Time PCR System (ROTOR-GENE).

### Statistical analysis

Statistical calculations were performed using the SPSS statistical software package (Version 13.0; SPSS, Inc. Chicago, IL). The measurement data were analyzed using one-way ANOVA. Statistical significance was considered for *P* < 0.05.

## Abbreviations

5-aza-dc: 5-aza-2′-deoxycytidine; ANOVA: analysis of variance; BALM: bi-asymmetric-Laplace model; BED: browser extensible data; BMP: bone morphogenetic protein; BSP: bisulfite sequencing PCR; CGI: CpG island; DMG: differently methylated gene; DMR: differently methylated region; EST: expressed sequence tag; FBS: fetal bovine syndrome; GO: gene ontology; KEGG: Kyoto Encyclopedia of Genes and Genomes; MSP: methylation-specific PCR; MSRE-qPCR: methylation sensitive restriction enzyme-based qPCR; PC: pancreatic cancer; PCR: polymerase chain reaction; PN: non-tumor tissue adjacent to pancreatic cancer; qPCR: quantitative polymerase chain reaction; RT-qPCR: reverse transcription quantitative polymerase chain reaction; TNM: tumor, node, and metastasis; TSS: transcription start site, UCSC, University of California, Santa Cruz

## Competing interests

The authors declare that they have no competing interests.

## Authors’ contributions

The experiments were conceived and designed by JY. The experiments were performed by YZ, JS, JG, WW, and XZ. Data were analyzed by SG, HZ, and NT. The paper was written by JY, YZ, and JS. All authors read and approved the final manuscript.

## Supplementary Material

Additional file 1: Figure S1 External and internal control DNA validation of MBD enrichment in a hypermethylated DNA fragment. **(A)** Spike DNA (containing fully methylated and unmethylated exogenous DNA fragments) was added to the pooled DNA samples. The methylated spike DNA appeared in the elution fraction containing more than 600 mM NaCl, and the unmethylated spike DNA appeared in the run-through fraction. This procedure was adopted to confirm the accuracy of methylated DNA enrichment in the present study. **(B)** Internal gene target control. As with the principle of spike DNA, internal gene targets that display a gradient of methylation statuses, such as *GAPDH* (unmethylated), *CFTR* (moderately methylated), and *TP63* (highly methylated), were used to evaluate the methylated DNA enrichment process. Here, we show that *GAPDH* rapidly eluted in the run-through fraction, *TP63* eluted in the 1000 mM NaCl fraction, and *CFTR* eluted in a fraction between these two extremes. Both the spike DNA and the internal control gene targets confirmed the accuracy of methylated DNA enrichment in this study. PC, pancreatic cancer; PN, non-tumor tissue adjacent to pancreatic cancer. Click here for file

Additional file 2: Table S1 PC-related hypermethylated DMR in orphan CGIs. Click here for file

Additional file 3: Table S2 PN-related hypermethtylated DMR in orphan CGIs. Click here for file

Additional file 4: Table S3 PC-related hypermethylated genes that lack CGIs in their promoters. Click here for file

Additional file 5: Table S4 PN-related hypermethylated genes that lack CGIs in their promoters. Click here for file

Additional file 6: Table S5 PC-related hyper-DMRs and the involved genes that were affected via both CGIs and CGI shore. Click here for file

Additional file 7: Table S6 PC-related hyper-DMRs and the involved genes that were affected via CGIs alone. Click here for file

Additional file 8: Table S7 PC-related hyper-DMRs and the involved genes that were affected via CGI shores alone. Click here for file

Additional file 9: Table S8 PN-related hyper-DMRs and the involved genes that were affected via both CGIs and CGI shores. Click here for file

Additional file 10: Table S9 PN-related hyper-DMRs and the involved genes that were affected via CGIs alone. Click here for file

Additional file 11: Table S10 PN-related hyper-DMRs and the involved genes that were affected via CGI shores alone. Click here for file

Additional file 12: Table S11 PC-related hypermethylated CGI in gene promoters. Click here for file

Additional file 13: Table S12 PN-related hypermethylated CGI in gene promoters. Click here for file

Additional file 14: Table S13 The effects of PC- and PN-related hypermethylated genes on tumorigenesis. Click here for file

Additional file 15: Table S14 MSP validation of 20 targets in clinical samples and cell lines. Click here for file

Additional file 16: Table S15 PC-related hypermethylated genes (loci) recovered by both methylCap-seq and microarray 244 k chip. Click here for file

Additional file 17: Table S16 PN-related hypermethylated genes (loci) recovered by both methylCap-seq and microarray 244 k chip. Click here for file

Additional file 18: Table S17 Gene symbol of genes aberrantly methylated in our study and previous studies. Click here for file

Additional file 19: Table S18 Analysis of aberrant methylation in pancreatic cancer by BSP and MSRE-qPCR. Click here for file

Additional file 20: Table S19 BSP, MSRE-qPCR, and MSP primers used in this study. Click here for file
