# Supplementary material for: High-frequency aberrantly methylated targets in pancreatic adenocarcinoma identified via global DNA methylation analysis using methylCap-seq
Source: Clin Epigenetics. 2014 Sep 22;6(1):18. doi: 10.1186/1868-7083-6-18 (PMC4177372; doi:10.1186/1868-7083-6-18)
Supplement: Additional file 1: Figure S1 — External and internal control DNA validation of MBD enrichment in a hypermethylated DNA fragment. (A) Spike DNA (containing fully methylated and unmethylated exogenous DNA fragments) was added to the pooled DNA samples. The methylated spike DNA appeared in the elution fraction containing more than 600 mM NaCl, and the unmethylated spike DNA appeared in the run-through fraction. This procedure was adopted to confirm the accuracy of methylated DNA enrichment in the present study. (B) Internal gene target control. As with the principle of spike DNA, internal gene targets that display a gradient of methylation statuses, such as GAPDH (unmethylated), CFTR (moderately methylated), and TP63 (highly methylated), were used to evaluate the methylated DNA enrichment process. Here, we show that GAPDH rapidly eluted in the run-through fraction, TP63 eluted in the 1000 mM NaCl fraction, and CFTR eluted in a fraction between these two extremes. Both the spike DNA and the internal control gene targets confirmed the accuracy of methylated DNA enrichment in this study. PC, pancreatic cancer; PN, non-tumor tissue adjacent to pancreatic cancer. [file 1868-7083-6-18-S1.pdf]

A

External Control

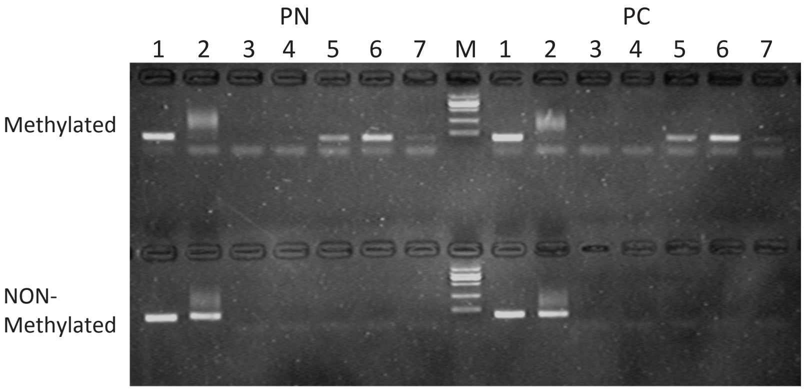

1. Positive Control
2. Non-captured DNA fraction(Run through)
3. 200 mM NaCl elution fraction
4. 450 mM NaCl elution fraction
5. 600 mM NaCl elution fraction
6. 1000 mM NaCl elution fraction
7. 2000 mM NaCl elution fraction

M. DL2000 Marker

B

Internal Control

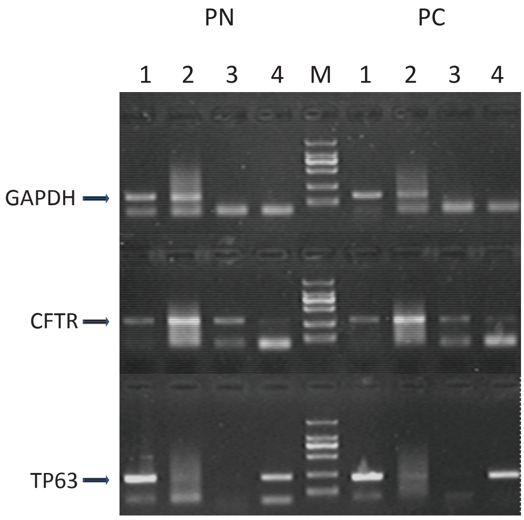

GAPDH denotes methylated regions of low density,CFTR denotes methylated regions of Medium density,TP63 denotes methylated regions of high density.

1. Positive Control
2. Non-captured DNA fraction(Run through)
3. 600 mM NaCl elution fraction
4. 1000 mM NaCl elution fraction
